# Post‐pubertal stability of split renal function after pyeloplasty performed in childhood

**DOI:** 10.1002/bco2.70159

**Published:** 2026-02-02

**Authors:** Mohamed A. Soltan, Tamer E. Helmy, Mohamed E. Dawaba, Mohamed Abou El‐Ghar, Ahmed Shokeir

**Affiliations:** ^1^ Urology and Nephrology Center Mansoura University Mansoura Egypt; ^2^ Department of Urology University of Tennessee Health Science Center Memphis Tennessee USA

**Keywords:** hydronephrosis resolution, late deterioration, paediatric pyeloplasty, post‐pubertal follow‐up, predictors of deterioration, renal function stability, renal pelvis reduction, split renal function, ureteropelvic junction obstruction

## Abstract

**Objectives:**

While early outcomes after pyeloplasty are well‐documented, long‐term split renal function (SRF) stability, particularly beyond puberty, remains unclear. This study evaluates SRF after prepubertal pyeloplasty and identifies factors associated with late deterioration.

**Methods:**

A retrospective review included paediatric patients who underwent pyeloplasty between 2000 and 2025. Exclusion criteria were solitary kidney, missing imaging, congenital anomalies or prior pyeloplasty. Patients were categorised into two groups: Group 1 (no deterioration) and Group 2 (late deterioration after puberty), based on ≥5% decrease in SRF. Clinical, surgical and imaging parameters were compared among groups using Chi‐square, Fisher's exact and Mann–Whitney *U* tests, with multivariate logistic regression to identify predictors of late deterioration.

**Results:**

Out of 1493 patients, 191 were included: 152 (79.6%) in Group 1 and 39 (20.4%) in Group 2. Group 2 patients were younger at surgery, had more frequent renal pelvis reduction and persistent hydronephrosis at 1‐year follow‐up (*p* < 0.001). Multivariate analysis identified renal pelvis reduction (odds ratio [OR] = 9.82, 95% confidence interval [CI]: 3.551–37.799, *p* < 0.001) and non‐resolution of hydronephrosis (OR = 4.811, 95% CI: 1.766–13.1, *p* = 0.002), as late deterioration predictors. Older age at surgery (OR = 0.958, 95% CI: 0.942–0.975, *p* < 0.001) and higher final SRF (OR = 0.905, 95% CI: 0.855–0.958, *p* < 0.001) were protective against deterioration.

**Conclusion:**

SRF may deteriorate after puberty despite initial post‐operative stability. Renal pelvis reduction, persistent hydronephrosis at 1 year and younger age at surgery are associated with increased risk of deterioration.

## 1 INTRODUCTION

Pyeloplasty is one of the most commonly performed procedures for paediatric urologic anomalies.^1^, ^2^ Although surgical techniques and perioperative management have become well‐standardised, questions remain regarding the appropriate follow‐up duration. Currently, no guidelines are available for duration or protocol of follow‐up after surgery.^3^ Significant differences persist among providers concerning follow‐up duration, protocols and the definition of treatment failure.^4^

Recurrence may manifest early after pyeloplasty through symptoms of obstruction, such as pain and febrile urinary tract infections (UTIs), which require urgent drainage of the affected kidney.^5^ In other cases, recurrence and failure can be indicated by an increase in the degree of hydronephrosis on ultrasound (US) or deterioration of split renal function (SRF) on renal scintigraphy, even in the absence of obvious symptoms.^5^, ^6^

After surgery, hydronephrosis may not resolve completely. As long as the patient's condition remains stable, additional surgery is generally not indicated. Decrease in anteroposterior diameter (APD) < 10 mm, percent of improvement of APD > 50%, has been considered resolution of hydronephrosis.^7^

It has been shown that changes in urine flow dynamics can lead to an increase in urinary system dilatation, forming the basis for using diuretic US. This imaging technique compares the degree of hydronephrosis before and after diuretic administration to assess whether the urinary system can accommodate increased urine output. An inability to do so signifies obstruction.^8^

Puberty is a period of substantial physiological changes, including significant increase in urine output compared to paediatric patients.^9^ This increase could place additional strain on the affected renal unit, potentially leading to a deterioration in SRF in patients who underwent unilateral pyeloplasty during childhood.^10^ As such, it is plausible that renal units with prior compromise may be vulnerable to functional decline during this period of rapid physiological change.

Long‐term follow‐up after pyeloplasty has been studied before; however, there has been a lack of inclusion of follow‐up after puberty specifically.^11^, ^12^, ^13^, ^14^ To our knowledge, there is only one study was conducted by Chertin et al. in 2009 that specifically included follow‐up after puberty.^10^

That retrospective study followed 49 children with prenatally diagnosed hydronephrosis who underwent pyeloplasty in infancy, concluding that renal function improved post‐operatively and remained stable throughout puberty, with only two patients requiring secondary intervention. This study had many limitations. The sample size was relatively small, and the cohort was restricted to a single centre over a short recruitment window (1989–1992). Imaging classifications such as Society of Foetal Urology (SFU) grade were retrospectively applied to archived US images, raising potential concerns about standardisation and accuracy. Furthermore, the study lacked a robust multivariable analysis of predictors for late deterioration and did not quantify the impact of surgical variables like renal pelvis reduction or post‐operative hydronephrosis resolution. Most critically, the outcome was reported as stable, despite 4% of the cohort undergoing reoperation. In our study. we aim to identify predictors of late deterioration; in addition, our sample size is larger.

Overall, this area remains underexplored in the literature. Our study aims to evaluate whether SRF remains stable after puberty in paediatric patients who underwent pyeloplasty before pubertal onset, and to identify clinical or surgical factors associated with late functional deterioration.

## 2 PATIENTS AND METHODS

### 2.1 Patients and inclusion criteria

Between 2000 and 2025, we retrospectively reviewed paediatric patients who underwent pyeloplasty prior to the onset of puberty at our institution. Eligible patients were those who had pyeloplasty for unilateral ureteropelvic junction obstruction (UPJO) and demonstrated no more than 5% deterioration in SRF on renal scintigraphy during the initial follow‐up period within 2 years after surgery.

### 2.2 Exclusion criteria

Patients were excluded if they were younger than 12 years old at their last follow‐up, had less than 5 years of postoperative follow‐up, or exhibited a decline in SRF greater than 5% within the first 2 years after surgery. Patients were also excluded if they had early post‐operative obstruction requiring urgent drainage, solitary kidney, bilateral UPJO, missing post‐operative renal scintigraphy (within 1–2 years or after puberty), missing pre‐operative imaging, lack of follow‐up imaging after puberty, associated congenital anomalies, such as vesicoureteral reflux or duplication anomalies, and any prior pyeloplasty performed at an outside institution.

### 2.3 Study design

Patients were classified into two groups based on post‐pubertal changes in SRF. Group 1 included patients with no deterioration in function, defined as stable SRF, improved SRF, or a decline in SRF of less than 5% compared to pre‐operative SRF. Group 2 included patients who experienced late deterioration, defined as a decline in SRF of 5% or more occurring at long‐term follow‐up after puberty.

### 2.4 Methods

Demographic and surgical data collected included age at surgery, sex, body mass index (BMI), laterality of surgery, presence of a crossing vessel, whether renal pelvis reduction was performed, use of ureteral stent, and presence of post‐operative urine leakage. Imaging studies were reviewed at three time points: pre‐operatively, early post‐operative follow‐up and late follow‐up after puberty. Early post‐operative follow‐up was identified as follow‐up within the first 2 years after surgery. Late follow‐up was defined as follow‐up performed only when both criteria were fulfilled: at least 5 years had elapsed since surgery and the patient had reached puberty. Renal scintigraphy was used to evaluate glomerular filtration rate (GFR) and SRF. US parameters assessed included the SFU grading system, which classifies hydronephrosis severity from grade 0 to 4. Grade 0 represents no dilatation; Grade 1 indicates a dilated renal pelvis without calyceal involvement; Grade 2 includes mild calyceal dilatation with normal parenchyma; Grade 3 refers to moderate dilatation of both the renal pelvis and calyces with preserved parenchymal thickness; and Grade 4 denotes severe pelvicalyceal dilatation accompanied by thinning of the renal parenchyma.^15^

Additional US parameters included the anteroposterior diameter (APD), which measures the maximal width of the renal pelvis in the transverse plane; parenchymal thickness, used as an indicator of preserved renal parenchyma; and the pelvis‐to‐cortex ratio, calculated by dividing the APD by the cortical thickness. Percent improvement in APD was defined as the percentage reduction in APD between pre‐operative and post‐operative measurements. Resolution of hydronephrosis was defined as either a reduction in APD greater than 50% or an SFU grade of 1 or lower.

### 2.5 Statistical analysis

Continuous variables were tested for normality using the Shapiro–Wilk test. All continuous data were found to be non‐normally distributed and are therefore presented as medians with interquartile ranges (IQRs). Categorical (nominal and ordinal) variables are expressed as frequencies and percentages. Comparisons between groups were performed using the Chi‐square test or Fisher's exact test for categorical variables, and the Mann–Whitney *U* test for continuous variables. Multivariate logistic regression analysis was used to identify independent predictors of late deterioration in split renal function, with results reported as odds ratios (ORs) and 95% confidence intervals (CIs). Given the collinearity among US parameters—specifically APD, parenchymal thickness and pelvis‐to‐cortex ratio—only the composite variable resolution of hydronephrosis was included in the regression model to avoid confounding because of multicollinearity. A two‐sided *p*‐value < 0.05 was considered statistically significant. Statistical analysis was performed using SPSS version 29 (IBM Corp., Armonk, NY).

### 2.6 Ethical consideration

Approval from the Institutional Review Board was obtained prior to study initiation. There were no conflicts of interest. This research did not receive any funding from public, commercial or not‐for‐profit sources.

## 3 RESULTS

**FIGURE 1:**
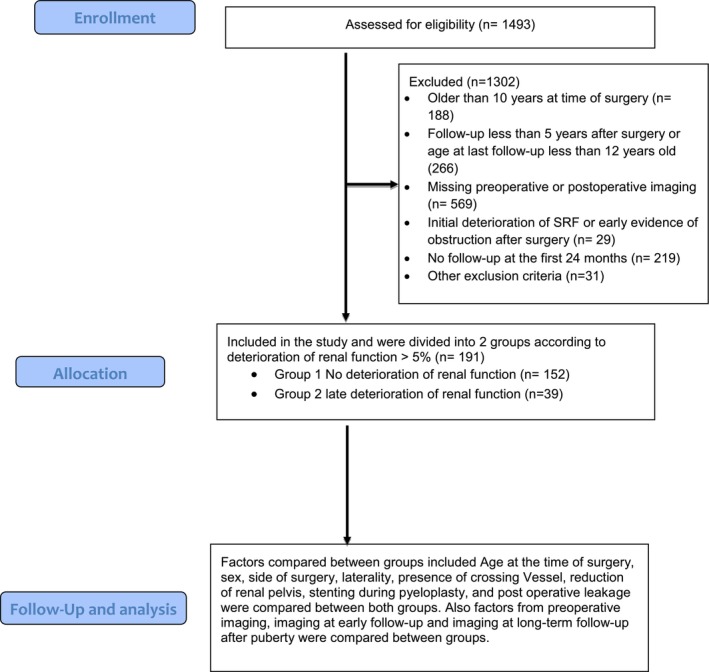
Patient selection flow chart.

A total of 1493 patients were screened for eligibility, of whom 1302 were excluded, leaving 191 patients included in the analysis (Figure 1). Patients were classified into two groups: Group 1 (no deterioration) included 152 patients (79.6%), and Group 2 (deterioration) included 39 patients (20.4%). Males were predominant in both groups, with no significant difference in sex distribution (*p* = 0.106). Patients in Group 2 were significantly younger at the time of surgery (median: 52 months) compared to Group 1 (median: 100 months; *p* < 0.001). BMI at surgery did not differ significantly between groups (*p* = 0.241) (Table 1).

**TABLE 1:** Patients demographics.

Variable	Group 1: no deterioration (no. = 152)	Group 2: deterioration (no. = 39)	*p*‐value
Sex, No. (%)			
• Male	96 (63.2%)	30 (76.9%)	0.106
• Female	56 (36.8%)	9 (23.1%)	
Median age at surgery in months (IQR)	100 (72–134)	52 (12–90)	**<0.001**
Median BMI at surgery (IQR), kg/m^2^	18.5 (16.8–20)	18.5 (17.3–22.2)	0.241
Median preop. GFR (IQR), ml/min	19 (14–32)	25 (16–26)	0.646
Median preop. SRF (IQR), %	33 (25–41)	41 (33–42)	0.0.09
Grades of hydronephrosis, no. (%)			
• Grade 0	0 (0%)	0 (0%)	0.648
• Grade 1	0 (0%)	0 (0%)	
• Grade 2	27 (17.8%)	9 (23.1%)	
• Grade 3	68 (44.7%)	18 (46.2%)	
• Grade 4	57 (37.5%)	12 (30.8%)	
Median preop. APD (IQR), cm	4.0 (3–5.3)	4.5 (3.7–4.6)	0.142
Median preop. parenchymal thickness (IQR), cm	0.75 (0.6–1)	0.9 (0.6–1)	0.096
Median preop. pelvis‐to‐cortex ratio (IQR)	5.1 (3.2–9)	4.1 (3.4–7.5)	0.274

Abbreviations: IQR, interquartile range; BMI, body mass index; preop, pre‐operative; GFR, glomerular filtration rate; SRF, split renal function; APD, anteroposterior diameter.

Pre‐operative functional and sonographic parameters including GFR, SRF, hydronephrosis grade, anteroposterior diameter (APD), parenchymal thickness and pelvis‐to‐cortex ratio were comparable between groups (Table 1).

Operative variables such as side of surgery, presence of a crossing vessel, use of a stent and immediate post‐operative leakage showed no significant differences between groups. However, renal pelvis reduction was performed significantly more often in Group 2 (38.5%) compared to Group 1 (4.6%) (*p* < 0.001) (Table 2).

**TABLE 2:** Operative data.

Variable, no. (%)	Group 1: no deterioration (no. = 152)	Group 2: deterioration (no. = 39)	*p*‐value
Side			
• Right	53 (34.9%)	11 (28.2%)	0.619
• Left	99 (65.1%)	28 (71.8%)	
Crossing vessel			
• Yes	20 (13.2%)	3 (7.7%)	0.423
• No	132 (86.8%)	36 (92.3%)	
Reduction of renal pelvis			
• Yes	7 (4.6%)	15 (38.5%)	**<0.001**
• No	145 (95.4%)	24 (61.5%)	
Stented			
• Yes	148 (97.4%)	39 (100%)	0.583
• No	4 (2.6%)	0 (0%)	
Immediate post‐operative leakage			
• Yes	3 (1.9%)	0 (0%)	1
• No	149 (98.1%)	39 (100%)	

**FIGURE 2:**
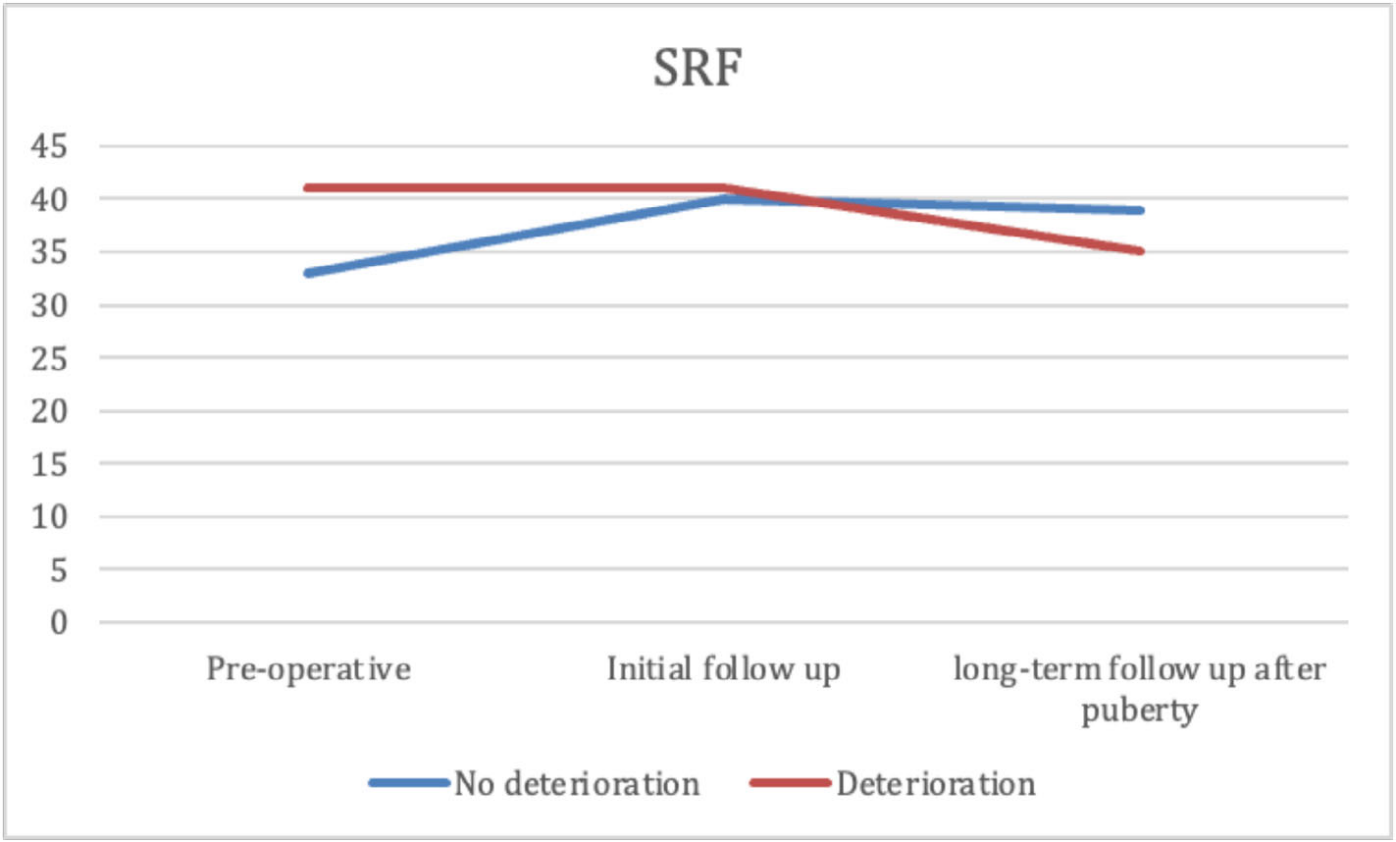
Split renal function median between groups.

**FIGURE 3:**
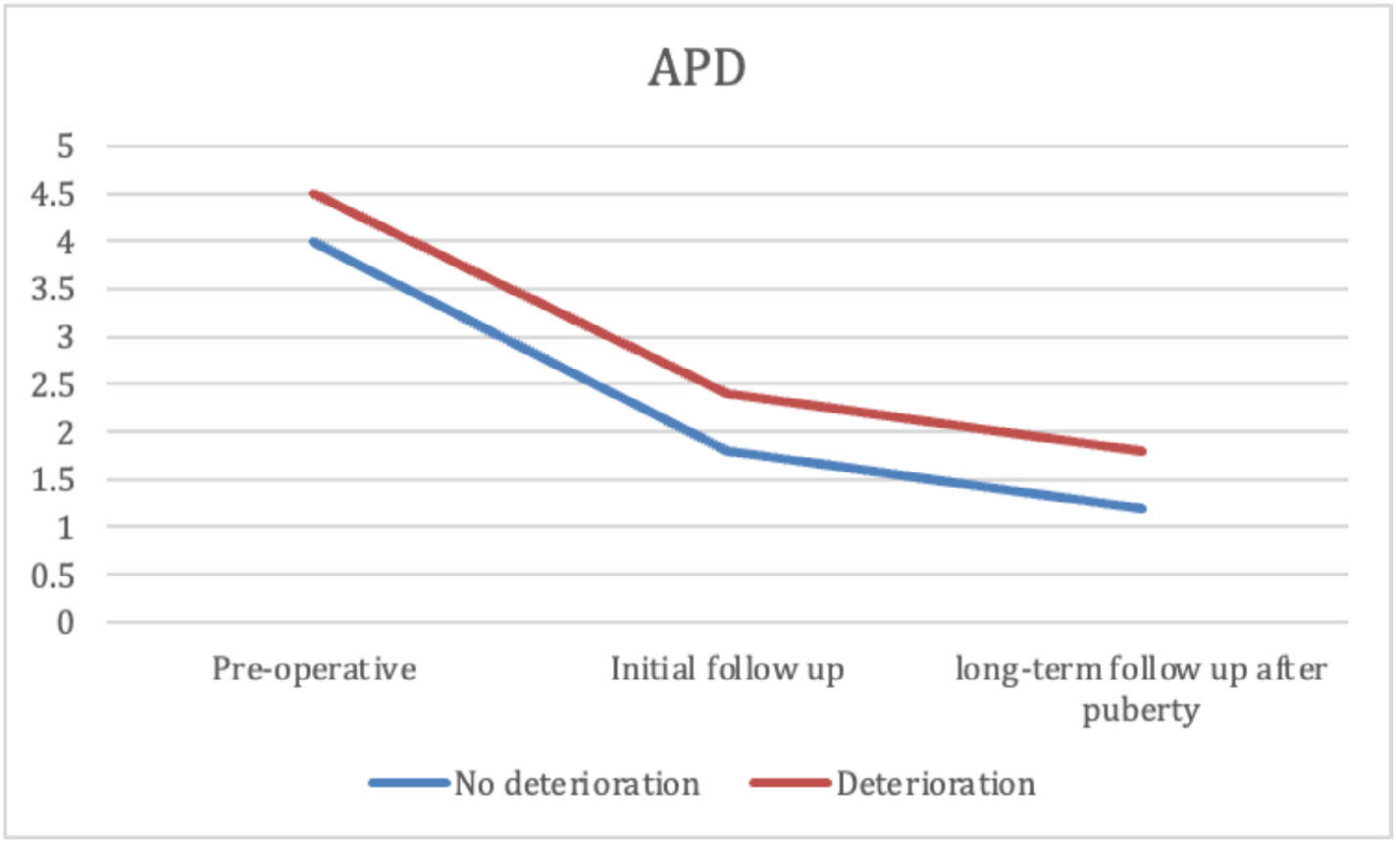
Antero‐posterior diameter median between groups.

**FIGURE 4:**
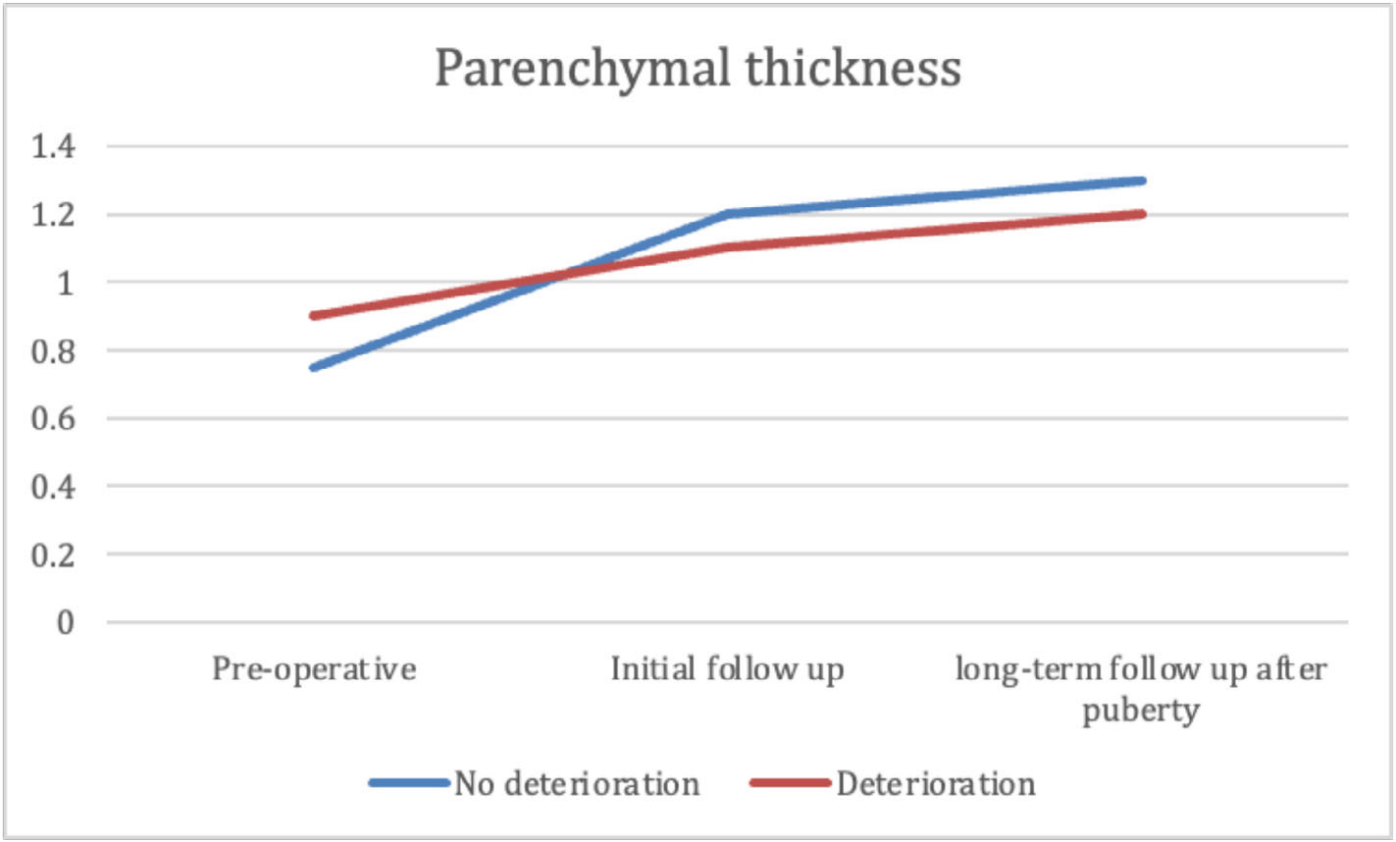
Parenchymal thickness median between groups.

**FIGURE 5:**
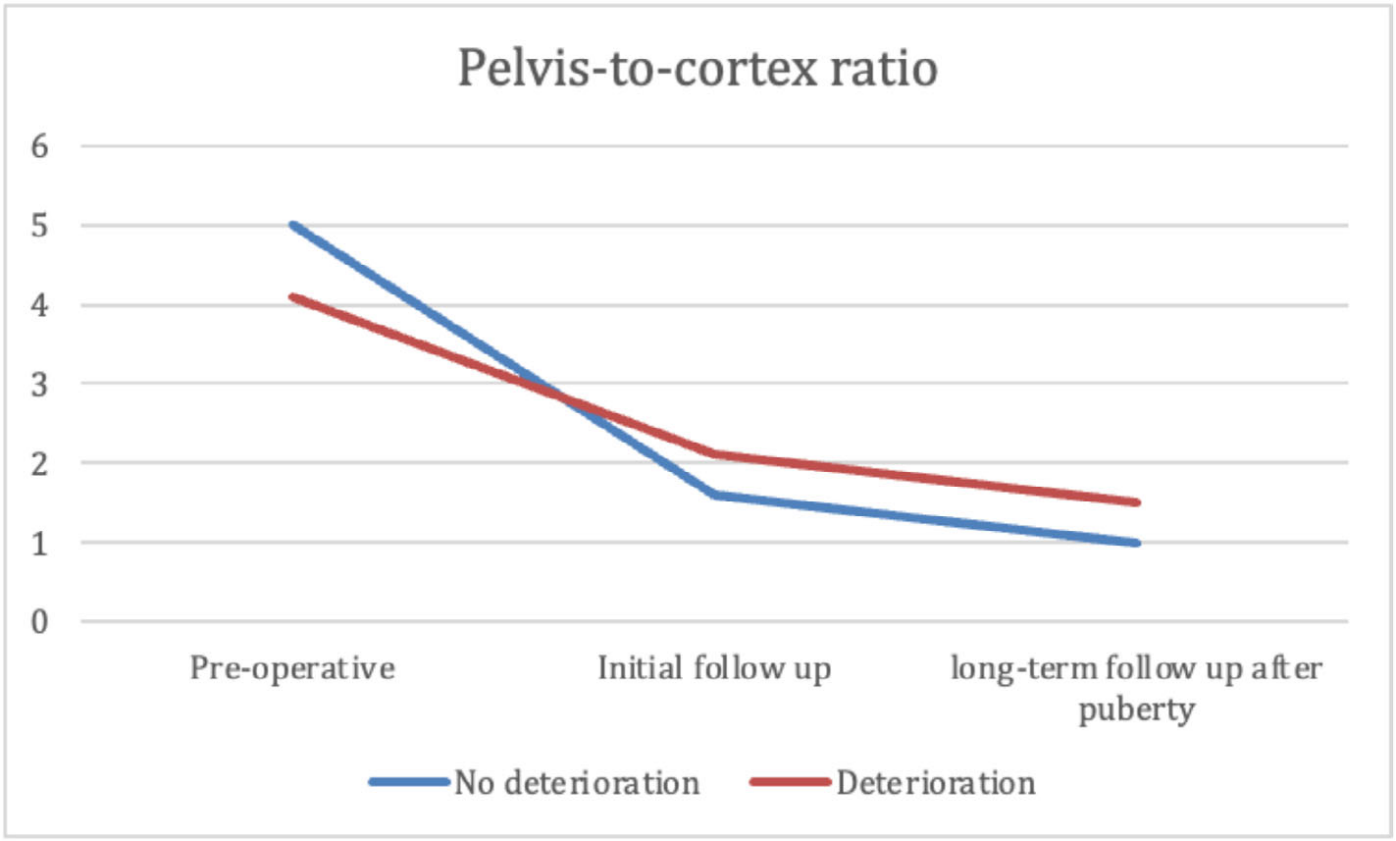
Parenchyma cortex ratio median between groups.

**FIGURE 6:**
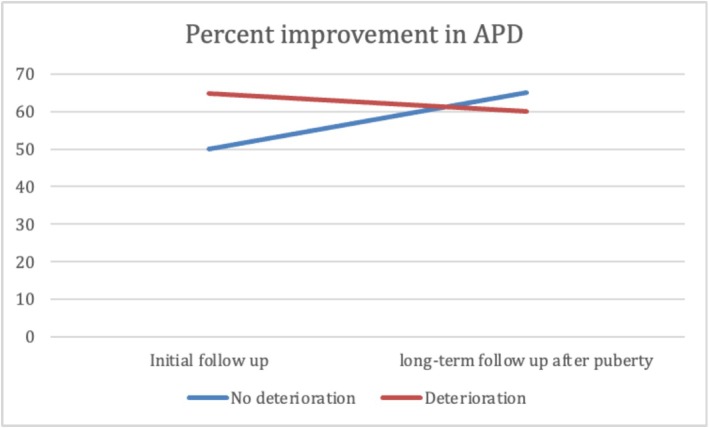
Percent improvement of APD diameter median between groups. APD, anteroposterior diameter.

**FIGURE 7:**
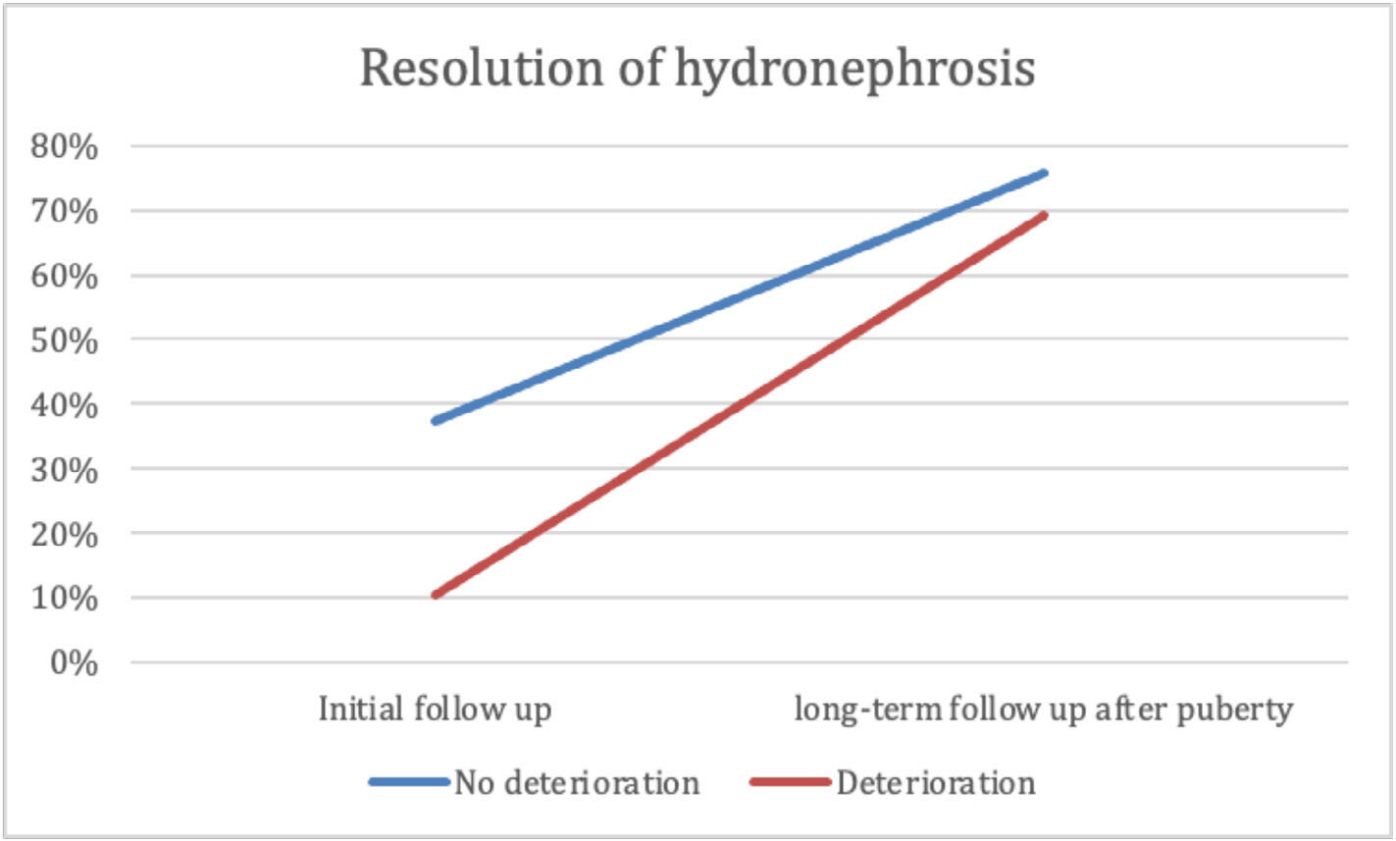
Resolution of hydronephrosis percentage between groups.

At the median age at initial follow‐up, patients in Group 2 were significantly younger than those in Group 1. No significant differences were observed between groups in GFR, SRF, or parenchymal thickness. However, Group 1 showed significantly better US findings, with more favourable SFU grades, smaller APD, lower pelvis‐to‐cortex ratio and higher percent improvement in APD. Additionally, resolution of hydronephrosis was significantly more common at initial follow‐up in Group 1 (37.5%) than in Group 2 (10.3%) (*p* < 0.001) (Table 3). At long‐term follow‐up after puberty, renal function remained significantly better in Group 1, with higher GFR and SRF values (*p* < 0.001). While hydronephrosis grades showed significant differences between the groups, there was no significant difference in APD, parenchymal thickness, pelvis‐to‐cortex ratio, percent improvement in APD or overall resolution between groups. (Table 3 and Figures 2, 3, 4, 5, 6, 7).

**TABLE 3:** Post operative follow‐up.

Variable	Group 1: no deterioration (no. = 152)	Group 2: deterioration (no. = 39)	*p*‐value
Initial follow‐up			
Median age at initial follow‐up in months (IQR)	113 (85.8–146)	60 (27–101)	**<0.001**
Median GFR at initial follow‐up (IQR), ml/min	27 (21–37)	25 (18–30)	0.059
Median SRF at initial follow‐up (IQR), %	40 (30–44)	41 (30–44)	0.872
Grades of hydronephrosis, No. (%)			
• Grade 0	0 (0%)	0 (0%)	**0.002**
• Grade 1	57 (37.5%)	3 (8.8%)	
• Grade 2	36 (23.7%)	16 (47.1%)	
• Grade 3	20 (14.5%)	8 (23.5%)	
• Grade 4	32 (24.3%)	7 (20.5%)	
Median APD at initial follow‐up (IQR), cm	1.8 (1.1–2.9)	2.4 (1.6–3.3)	0.**002**
Median parenchymal thickness at initial follow‐up (IQR), cm	1.2 (0.9–1.3)	1.1 (0.8–1.3)	0.85
Median pelvis‐to‐cortex ratio at initial follow‐up (IQR)	1.6 (0.9–2.5)	2.1 (1.3–3.4)	**0.007**
Median percent improvement in APD at initial follow‐up (IQR), %	50.0 (29.5–73.42)	34.8 (26.7–54.8)	**0.023**
Resolution 1 year, No. (%)			
• Yes	57 (37.5%)	4 (10.3%)	**<0.001**
• No	95 (62.5%)	35 (89.7%)	
Long‐term follow‐up after puberty			
Median age at late follow‐up (IQR)	197 (175–223)	181 (153–204)	0.105
Median GFR at long‐term follow‐up (IQR), ml/min	33.5 (22.8–44.3)	23 (19.35)	**<0.001**
Median SRF at long‐term follow‐up (IQR), %	39 (31–44)	35 (28–37)	**<0.001**
Grades of hydronephrosis, no. (%)			
• Grade 0	4 (2.6%)	0 (0%)	**<0.001**
• Grade 1	111 (73%)	27 (69.2%)	
• Grade 2	27 (17.8%)	0 (0%)	
• Grade 3	0 (0%)	5 (12.8%)	
• Grade 4	10 (6.6%)	7 (18%)	
Median APD at long‐term follow‐up (IQR), cm	1.2 (0.8–2.4)	1.8 (1–2.7)	0.179
Median parenchymal thickness at long‐term follow‐up (IQR), cm	1.3 (1.1–1.5)	1.2 (1–1.5)	0.491
Median pelvis‐to‐cortex ratio at long‐term follow‐up (IQR)	0.98 (0.58–1.98)	1.5 (0.71–2.45)	0.157
Median percent improvement in APD at long‐term follow‐up (IQR), %	64.91 (40.7–77.8)	60.0 (37.8–75.6)	0.617
Resolution at long‐term follow‐up, no. (%)			
• Yes	115 (75.7%)	27 (69.2%)	0.412
• No	37 (24.3%)	12 (30.8%)	

Abbreviations: IQR, interquartile range; BMI, body mass index; preop, pre‐operative; GFR, glomerular filtration rate; SRF, split renal function; APD, anteroposterior diameter.

Given the collinearity between sonographic variables such as APD, parenchymal thickness, pelvis‐to‐cortex ratio and percent improvement in APD, only resolution of hydronephrosis was included in the multivariate logistic regression to avoid confounding because of multicollinearity. Similarly, age at first follow‐up is closely related to age of surgery as it is defined as follow‐up within first 2 years after surgery, so only age at surgery was included in the multivariate analysis. On multivariate logistic regression analysis, reduction of the renal pelvis was associated with a significantly increased risk of late deterioration (OR = 9.82, 95% CI: 3.551–37.799, *p* < 0.001). Lack of hydronephrosis resolution at initial follow‐up was also predictive of late deterioration (OR = 4.811, 95% CI: 1.766–13.1, *p* = 0.002). In contrast, older age at surgery (OR = 0.958, 95% CI: 0.942–0.975, *p* < 0.001) and higher final SRF on renogram (OR = 0.905, 95% CI: 0.855–0.958, *p* < 0.001) were protective against deterioration (Table 4).

**TABLE 4:** Multivariate analysis comparison between Group 1 and Group 2.

	Odds ratio	95% C.I.		*p*‐value
Reduction of renal pelvis	9.82	3.551	37.7989	<0.001
Nonresolution of hydronephrosis at 1 year of follow‐up	4.811	1.766	13.108	0.002
Age at surgery	0.958	0.942	0.975	<0.001
Last renogram split renal function	0.905	0.855	0.958	<0.001

## 4 DISCUSSION

Success rates of pyeloplasty in various reports showed different numbers from 88% to 100%.^3^, ^5^, ^6^, ^16^, ^17^, ^18^, ^19^, ^20^ In a large meta‐analysis including 10 studies and 3494 patients, the recurrence rate was <4% for both open and minimal invasive surgery pyeloplasty.^20^ In our study, deterioration groups are 39, representing about 20.4% of the study population. However, this percentage is not accurate, given the strict inclusion criteria possibly creating attrition bias because patients who generally do well tend to be lost at long‐term follow‐up. Most patients who were excluded from the study had initial follow‐up and no long‐term follow‐up. In this case, the comparison should be total deterioration with the initial number of patients included in the study, which is 1493; the percentage will be 2.6%.

In addition, late deterioration in our cohort may reflect limited growth potential of a previously obstructed renal unit. A renal unit with a history of obstruction may not grow proportionately with its contralateral kidney, resulting in a relative decline in SRF despite stable drainage. Most prior studies evaluated only early post‐operative outcomes or late outcomes without extending surveillance beyond puberty. Thus, the observed 20.4% reflects a selected long‐term follow‐up cohort influenced both by attrition bias and by the renal unit's relative growth potential, rather than representing the overall post‐operative pyeloplasty population.

Our previous data from 2009 showed a failure rate of 3%.^6^ In this study, the definition of failure was reoperation, not just the decrease in SRF, and varies depending on multiple clinical and radiological factors. In our study, we only included patients with initial success and then classified them according to the SRF change from baseline at long‐term follow‐up. Many studies recommended the use of >5% of SRF as the cut for definition of deterioration.^12^, ^13^, ^21^

The study by van den Hoek et al. suggests that long‐term follow‐up after paediatric pyeloplasty may not be necessary, as most patients in their cohorts showed stable SRF for 5–7 years post‐operatively. However, their conclusions are based on only 29 patients with long‐term follow‐up, of whom two (7%) experienced significant SRF deterioration, indicating that late functional decline is possible. Additionally, their follow‐up period did not extend beyond puberty, leaving uncertainty regarding potential changes in renal function during adolescent growth. This contrasts with our study, which highlights that even patients with initial improvement in hydronephrosis on US can still experience late SRF deterioration after puberty.^11^

Also another study showed no long‐term deterioration after 3 and 6 months of follow‐up if remain stable.^12^ This study only included 28 patients with one patient developing early deterioration and all other patients showed stable SRF at long‐term follow‐up. The long‐term follow‐up in this study was defined as >5 years with median of 10 years. However, it did not specifically mention follow‐up after puberty and has small sample size.

In 2007, Matsumoto et al. concluded that SRF was not stable in some of their study population at long‐term follow‐up after initial improvement. This aligns with our findings; however, this study focussed only on long‐term follow‐up and not necessarily post pubertal.^13^

The only study—to our knowledge—that studied the effect of puberty on SRF after paediatric pyeloplasty was conducted in 2009. In this study, 49 patients were included in the study and two of them required endopyelotomy 8 and 10 years after pyeloplasty, representing about 4% of the population. The authors concluded that SRF remains unchanged after puberty.^10^ The sample size in this study was small, and yet, there has been reoperation in 4% after initial follow‐up that was stable.

According to a study by Dy et al. (2016), most of the deterioration of renal function happens in the early post‐operative period. In this study, it was shown that while most secondary procedures after paediatric pyeloplasty occur within the first year (87.2%), a smaller but significant portion (8.4%) happens between 1 and 2 years, and 4.4% even later. Management included stent or drain placement, endoscopic procedures and redo pyeloplasty, with a few cases requiring nephrectomy or even transplantation. This highlights that although early failures are more common, late failures can still occur, reinforcing the need for continued follow‐up beyond the initial post‐operative period.^14^

A study by Park et al. underscores the long‐term nature of hydronephrotic changes after pyeloplasty, with a follow‐up extending into adulthood. In this study, there was no renal scintigraphy performed in long‐term follow‐up. Relying only on US, this study found stabilisation to improvement of hydronephrosis after a median time to initial improvement of 8 months and full resolution taking over 3 years. In this study, once improvement occurred, no late recurrence was observed. In our study, improvement was noted not only with no deterioration but also with late deterioration group. This highlights that in our study, early improvement of US was associated with long‐term improvement of US and not necessarily stabilisation of SRF. In addition, our study included only patients who were post‐pubertal during follow‐up, unlike this study, which showed long‐term follow‐up not necessarily post‐pubertal.^22^

The term resolution of hydronephrosis has been used before in literature to categorise patients at follow‐up. While it is typically not expected for the kidney to look totally normal after pyeloplasty, it has been postulated that APD less than 1 cm or >50% decrease in APD as resolution of hydronephrosis.^14^ We added that SFU grade can be 1 or less to this and considered these parameters as resolution of hydronephrosis in our study. Multiple US parameters were postulated in the follow‐up of hydronephrosis. In our study, these factors were studied separately; however, in multivariate analysis, we only included the resolution of hydronephrosis for simplification of statistics. Also, because it is closely related to the other parameters of US and can lead to multicollinearity and alter the effect of US in follow‐up, rendering it non‐significant because variables are closely related. Initial resolution was significantly higher in the no deterioration group, giving the impression that initial follow‐up can help in identification of patients who are at risk of later deterioration of hydronephrosis.

Most of our patient population are males, which aligns with literature.^23^, ^24^, ^25^ In our study, the male patients showed no difference between both groups. Some studies showed no difference between males and females in the success of pyeloplasty.^14^, ^26^ Another study showed an association between male sex and the need for reoperation following pyeloplasty; however, no difference at post‐operative resolution.^25^

Although our analysis showed an association between renal pelvis reduction and late deterioration, this finding should not be interpreted as a cause of failure. Previously, reduction of the renal pelvis during pyeloplasty showed no effect on the outcome.^27^ In our study, pelvic reduction was a subjective intraoperative decision made in case of marked dilatation. The extent of reduction was not standardised or quantified. Therefore, the need for reduction likely reflects pre‐operative anatomical severity, not a cause of deterioration. Thus, renal pelvis reduction should be viewed as a marker of severe UPJO, not a risk factor for deterioration.

Although our analysis demonstrated an association between renal pelvis reduction and late deterioration, this should not be interpreted as a procedural cause of failure. Prior work showed that pelvic reduction does not affect surgery outcome.^27^ In our cohort, the decision of pelvic reduction was subjective, performed only in cases of marked dilation, and the degree of reduction was neither standardised nor quantified. Thus, the need for reduction likely reflects pre‐operative anatomical severity rather than contributing to deterioration. Renal pelvis reduction should be viewed as a marker of severe UPJO, not an independent risk factor for late functional decline.

In our cohort, younger age at surgery was statistically associated with late deterioration; however, this effect was small (OR 0.958) and is best interpreted as a surrogate marker of baseline disease severity rather than an adverse effect of early intervention. Children who undergo pyeloplasty in infancy typically present with more severe obstruction and may have limited nephron reserve at baseline. In addition, relative SRF decline may simply reflect limited growth potential of the renal unit with history of obstruction rather than recurrent obstruction. Therefore, the observed association does not contradict the established benefits of early repair; rather, it highlights that a decline in SRF over time may reflect differential renal growth rather than recurrent obstruction.

While some studies tried to explore standardised follow‐up protocol,^28^ the follow‐up remains variable among various studies. In our study, we could identify late deterioration in asymptomatic patients after puberty. Limitations of the study are being retrospective in nature. Also, heterogeneity of imaging studies that have been performed over a long period of time makes it sometimes difficult to compare.

## 5 CONCLUSION

Most patients who undergo pyeloplasty in childhood tend to have no deterioration of renal function after puberty. However, deterioration of renal function after pyeloplasty may occur in some patients, even those with initially favourable outcomes. Reduction of the renal pelvis, persistent hydronephrosis at 1 year and younger age at surgery were identified as significant predictors of late functional decline. These findings can guide during follow‐up of these patients beyond puberty. Further prospective studies are warranted to validate these predictors and guide follow‐up protocols.

## AUTHOR CONTRIBUTIONS

**Mohamed A. Soltan:** Study design, data collection, data analysis and manuscript writing. **Tamer E. Helmy:** Idea of the manuscript, writing manuscript and statistical analysis. **Mohamed E. Dawaba:** Revision of the final manuscript. **Mohamed Abou El‐Ghar:** Revision of the manuscript and data collection. **Ahmed Shokeir:** Design and implementation of the research and revising final manuscript.

## CONFLICT OF INTEREST STATEMENT

The authors declare no conflicts of interest.
